# Cytokine Consistency Between Bone Marrow and Peripheral Blood in Patients With Philadelphia-Negative Myeloproliferative Neoplasms

**DOI:** 10.3389/fmed.2021.598182

**Published:** 2021-06-24

**Authors:** Pu Chen, Boting Wu, Lili Ji, Yanxia Zhan, Feng Li, Luya Cheng, Jingjing Cao, Hehui Chen, Yang Ke, Zhihui Min, Lihua Sun, Fanli Hua, Hao Chen, Yunfeng Cheng

**Affiliations:** ^1^Department of Laboratory Medicine, Zhongshan Hospital, Fudan University, Shanghai, China; ^2^Department of Transfusion Medicine, Zhongshan Hospital, Fudan University, Shanghai, China; ^3^Department of Hematology, Zhongshan Hospital, Fudan University, Shanghai, China; ^4^Department of Hematology, Zhongshan Hospital Qingpu Branch, Fudan University, Shanghai, China; ^5^Institute of Clinical Science, Zhongshan Hospital, Fudan University, Shanghai, China; ^6^Department of Thoracic Surgery, Zhongshan Hospital Xuhui Branch, Fudan University, Shanghai, China; ^7^Center for Tumor Diagnosis & Therapy, Jinshan Hospital, Fudan University, Shanghai, China

**Keywords:** philadelphia-negative myeloproliferative neoplasms, bone marrow, peripheral blood, microenvironment, cytokine

## Abstract

**Background:** Inflammation might play a critical role in the pathogenesis and progression of Philadelphia-negative myeloproliferative neoplasms (Ph^−^MPNs) with elevated inflammatory cytokines in peripheral blood (PB). However, the inflammatory status inside the bone marrow (BM), which is the place of malignancy origin and important microenvironment of neoplasm evolution, has not yet been elucidated.

**Methods:** Inflammatory cytokine profiles in PB and BM of 24 Ph-MPNs patients were measured by a multiplex quantitative inflammation array. Cytokines that correlated between PB and BM were selected and then validated by ELISA in a separate cohort of 52 MPN patients. Furthermore, a panel of cytokines was identified and examined for potential application as non-invasive markers for the diagnosis and prediction of fibrosis progress of MPN subtypes.

**Results:** The levels of G-CSF, I-309, IL-1β, IL-1ra, IL-12p40, IL-15, IL-16, M-CSF, MIG, PDGF-BB, and TIMP-1 in BM supernatants were significantly higher than those in PB (all *p* < 0.05). Linear correlations between BM and PB levels were found in 13 cytokines, including BLC, Eotaxin-2, I-309, sICAM-1, IL-15, M-CSF, MIP-1α, MIP-1δ, RANTES, TIMP-1, TIMP-2, sTNFRI, and sTNFRII (all R > 0.4 and *p* < 0.05). Levels of BLC, Eotaxin-2, M-CSF, and TIMP-1 in PB were significantly different from those in health controls (all *p* < 0.05). In PB, levels of TIMP-1 and Eotaxin-2 in essential thrombocythemia (ET) group were significantly lower than those in groups of prefibrotic primary myelofibrosis (pre-PMF) [TIMP-1: 685.2 (322.2–1,229) ng/ml vs. 1,369 (1,175–1,497) ng/ml, *p* = 0.0221; Eotaxin-2: 531.4 (317.9–756.6) pg/ml vs. 942.4 (699.3–1,474) pg/ml, *p* = 0.0393] and primary myelofibrosis (PMF) [TIMP-1: 685.2 (322.2–1229) ng/ml vs. 1,365 (1,115–1,681) ng/ml, *p* = 0.0043; Eotaxin-2: 531.4 (317.9–756.6) pg/ml vs. 1,010 (818–1,556) pg/ml, *p* = 0.0030]. The level of TIMP-1 in myelofibrosis (MF) >1 group was significantly higher than that in MF ≤ 1 group.

**Conclusion:** Abnormal inflammatory status is present in MPN, especially in its BM microenvironment. Consistency between PB and BM levels was found in multiple inflammatory cytokines. Circulating cytokine levels of BLC, M-CSF, Eotaxin-2, and TIMP-1 reflected inflammation inside BM niche, suggesting potential diagnostic value for MPN subtypes and prognostic value for fibrosis progression.

## Introduction

The classical myeloproliferative neoplasms (MPNs), also called Philadelphia-negative myeloproliferative neoplasms (Ph-MPNs), namely essential thrombocythemia (ET), polycythemia vera (PV), and primary myelofibrosis (PMF), are malignant hematologic disease originating from stem cells (1). These diseases often have an occult and similar clinical onset, with a lengthy and progressive course. PV and ET patients may develop secondary myelofibrosis or even progress to acute myeloid leukemia (AML). Recent evidence showed that sustained inflammation plays a crucial role in the pathogenesis and progression of Ph-MPNs with series of elevated levels of inflammatory cytokines present in peripheral blood (PB) (2–4). The newly launched *JAK2* inhibitors were found to have a significant effect in improving clinical symptoms in MPN patients without *JAK2* mutations by regulating excessive immune response (5). Once a key driver gene mutation such as *JAK2* V617F, *CALR*, or *MPL* occurs, clonal hematopoietic cells and related stromal cells may affect chromosomal stability and promote secondary clonal evolution via secreting a series of inflammatory cytokines during proliferation, resulting in heterogeneity of clinical manifestations. Bone marrow (BM) niche is a relatively barriered tumor microenvironment, which plays an important role in maintaining hematopoietic stem cells. Manshouri et al. reported that cytokines secreted by BM stromal cells have a protective effect on clonal hematological malignant cells in *in vitro* co-culture experiments (6). Koen Schepers et al. proposed that myeloid malignant cells can stimulate mesenchymal cells to overproduce functionally altered osteoblastic lineage cells (OBC) (7). These OBCs accumulate in the BM cavity as inflammatory myelofibrotic cells, which favors the proliferation of leukemic stem cells. However, the inflammatory state inside the BM niche has not been investigated in MPN yet. Due to the indolent onset, similar clinical manifestations, and long progression course, it brings great challenges to the early differential diagnosis of different MPN subtypes, as well as disease progression monitoring. As such, the present study aims to investigate the characteristics of inflammatory cytokines in both BM niche and PB of MPN patients, and to explore their potential clinical applications.

## Materials and Methods

### Study Subjects

All MPN patients enrolled in the present study were diagnosed in our institution based on the diagnostic criteria established by WHO classification of tumors of hematopoietic and lymphoid tissues (2016 revision) (1) from January 2018 to February 2020. *JAK2* V617F, *JAK2* EXON12, *MPL* W515L/K, *CALR* mutations, and *BCR-ABL* fusion gene were detected via MPN-related gene detection kit based on PCR probes (YUANQI Co, Ltd, Shanghai, China). *BCR-ABL* positive (chronic myeloid leukemia, CML) patients were excluded. None of these patients were on active therapy when samples were collected. The health control group comprised 36 gender- and age-matched healthy volunteers who volunteered to provide PB samples during the same study period.

Approximate 2 ml of BM and 3 ml PB were collected from each patient simultaneously using EDTA tubes (Becton, Dickinson and Company; Franklin Lakes, NJ, USA). BM supernatants and PB plasma were obtained and immediately stored at −80°C until test.

MF grading was identified by silver staining of BM biopsy sections according to 2016 WHO grading system (MF-0: with no increase in reticulin. MF-1: with a very loose network of reticulin fibers. MF-2: showing a more diffuse and dense increase in reticulin fibers and some coarse collagen fibers. MF-3: with coarse bundles of collagen fibers intermingled with dense reticulin, accompanied by initial osteosclerosis).

The study was conducted in accordance with the Declaration of Helsinki, study protocol was approved by our Institutional Review Board, written informed consent was obtained from each participant or their guardian upon enrollment.

### Inflammatory Cytokine Array

Paired BM supernatants and PB plasma from MPN patients, and PB plasma from control group were measured for inflammatory cytokine profiles using Quantibody Human Inflammatory Array 3 (RayBiotech, Norcross, GA, USA) which could detect 40 inflammatory cytokines simultaneously, including I-309 (CCL1), sICAM-1 (CD54), IFN-γ, MCP-1 (CCL2), MIP-1α (CCL3), MIP-1β (CCL4), MIP-1δ (CCL15), RANTES (CCL5), Eotaxin-1 (CCL11), Eotaxin2 (CCL24), MIG (CXCL9), BLC (CXCL13), G-CSF, M-CSF, GM-CSF, IL-1α, IL-1β, IL-1ra, IL-2, IL-4, IL-5, IL-6, IL-6sR, IL-7, IL-8 (CXCL8), IL-10, IL-11, IL-12p40, IL-12p70, IL-13, IL-15, IL-16, IL-17A, PDGF-BB, TIMP-1, TIMP-2, TNFα, TNFβ, sTNFRI, and sTNFRII. Per manufacturer's protocol, thawed samples were added into the protein array pools spotted by specific capture antibodies and incubated overnight. Four replicate wells were made for each sample, and some samples were pre-diluted if necessary. After extensive washing, the arrays were incubated with a cocktail of biotinylated antibodies. The array slides with bound biotin were then incubated with streptavidin conjugated Hylite Plus 555 fluor. Relative fluorescent strength was detected by LuxScan 10 K-A Microarray Scanner (CapitalBio Corporation, Beijing, China). Signal values were captured with Mapix software. The actual concentration of every protein was analyzed and obtained by standard curve plotted via standard controls from the array.

### Enzyme Linked Immunosorbent Assay (ELISA)

Cytokines whose PB levels are significantly different to that of control group (*p* < 0.05), and also have optimal correlation with BM levels (*R* > 0.6, *p* < 0.05) were identified and selected based on the results of cytokine array. The selected cytokines were examined by ELISA kits (Ray Biotech., Norcross, GA, USA) in plasma samples of a separate cohort consisting of 52 Ph-MPN patients and 20 health volunteers. The procedure was performed following the manufacturer's protocol. Absorbance was measured at a wavelength of 450 nm using a microplate reader (ST-360, Kehua Bioengineering Co., Shanghai, China). Finally, cytokine concentration was obtained on standard curve fitted using ELISA Calc software.

### Statistical Analyses

Statistical analysis was performed with the SPSS 19.0 software for windows (SPSS, Chicago, IL, USA). All quantitative data are expressed as mean ± standard deviation (for normal distribution) or medians (inter-quartile ranges) (for non-normal distribution). The correlation of cytokine levels between BM supernatant and PB plasma was tested by Pearson's correlation coefficient or Spearman rank correlation coefficient, as appropriate. Comparisons among the groups were performed by one-way ANOVA with the least significant difference test for test for *post-hoc* multiple comparisons, Kruskal-wallis test and Mann-Whitney *U*-test as appropriate. Statistical significance was defined as two-sided *p* < 0.05.

## Results

### Paired Profiling of PB and BM Cytokines of MPN

**Figure 1:**
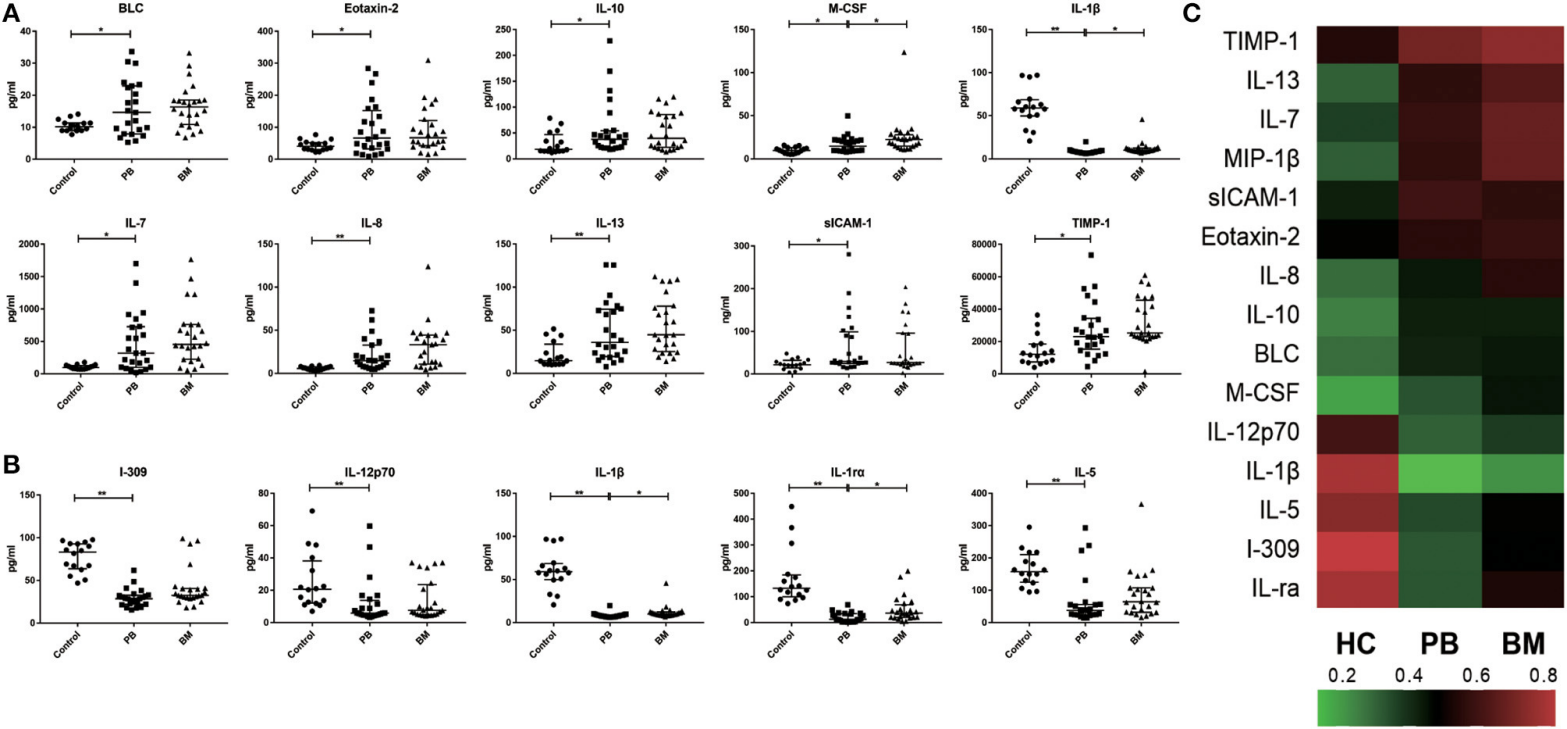
Characteristics of inflammatory cytokines in PB and BM of MPN patients. **(A)** The plasma levels of BLC, Eotaxin-2, IL-7, IL-8, IL-13, IL-10, M-CSF, MIP-1β, sICAM-1, TIMP-1 in MPN were significantly higher than those in health control group; **(B)** The plasma levels of IL-12p70, IL-1ra, IL-1β, IL-5, I-309 in MPN were significantly lower than those in control group; **(C)** Heat maps of overall characteristics of 15 cytokines with significant differences in PB levels between MPN patients and control group. (^*^p < 0.05; ^**^p < 0.01).

A total of 24 MPN patients (4 with PV, 13 with ET, 3 with Pre-PMF and 4 with PMF) were included in the paired profiling of cytokine array investigation. Levels of BLC [14.66 (7.95–22.95) pg/ml vs. 10.17 (8.97–11.34) pg/ml, *p* = 0.0423], Eotaxin-2 [66.47 (32.63–152.30) pg/ml vs. 40.96 (30.25–52.62) pg/ml, *p* = 0.0467], sICAM-1 [29.78 (24.94–98.98) × 10^3^ pg/ml vs. 21.94 (14.44–32.21) × 10^3^ pg/ml, *p* = 0.0355], IL-7 [318.10 (100.90–727.00) pg/ml vs. 96.10 (74.66–127.40) pg/ml, *p* = 0.0111], IL-8 [14.15 (7.60–32.83) pg/ml vs. 5.71 (4.02–7.67) pg/ml, *p* = 0.0005], IL-10 [37.82 (21.82–54.32) pg/ml vs. 18.74 (14.86–47.03) pg/ml, *p* = 0.0419], IL-13 [35.98 (19.62–74.48) pg/ml vs. 14.83 (10.68–33.83) pg/ml, *p* = 0.0088], M-CSF [14.67 (9.34–21.70) pg/ml vs. 9.65 (6.37–12.97) pg/ml, *p* = 0.0498], MIP1β [60.36 (25.85–116.20) pg/ml vs. 11.40 (4.36–82.85) pg/ml, *p* = 0.0199], and TIMP-1 [23.01 (15.29–34.32) × 10^3^ pg/ml vs. 12.16 (7.38–18.41) × 10^3^ pg/ml, *p* = 0.0205] in plasma of MPN patients were significantly higher than those of the control group (Figure 1A), while levels of I-309 [28.59 (22.24–32.76) pg/ml vs. 83.31 (63.73–92.99) pg/ml, *p* < 0.0001], IL-12p70 [5.91 (4.61–13.81) pg/ml vs. 20.70 (12.11–38.20) pg/ml, *p* = 0.0008], IL-1β [7.75 (6.75–8.82) pg/ml vs. 59.00 (49.86–68.50) pg/ml, *p* < 0.0001], IL-1ra [12.58 (2.89–34.59) pg/ml vs. 133.20 (99.74–184.20) pg/ml, *p* < 0.0001], and IL-5 [37.98 (24.57–56.14) pg/ml vs. 157.40 (124.90–210.50) pg/ml, *p* < 0.0001] were lower than those of the control group (Figure 1B). Among these significantly differenced cytokines in MPN patients, the levels of M-CSF [14.67 (9.34–21.70) pg/ml vs. 25.24 (14.81–27.90) pg/ml, *p* = 0.0323], IL-1rα [12.58 (2.89–34.59) pg/ml vs. 36.68 (20.03–68.73) pg/ml, *p* = 0.0264), and IL-1β (7.75 (6.75-8.82) pg/ml vs. 9.92 (8.52–12.38) pg/ml, *p* = 0.014] in plasma were significantly lower than their corresponding levels in BM. Moreover, except for sICAM-1, the BM levels of all the rest cytokines were numerically higher than their corresponding plasma levels, although no significant statistical differences were reached (Figure 1C).

### Comparison of Cytokines in BM and PB in MPN Patients

**Figure 2:**
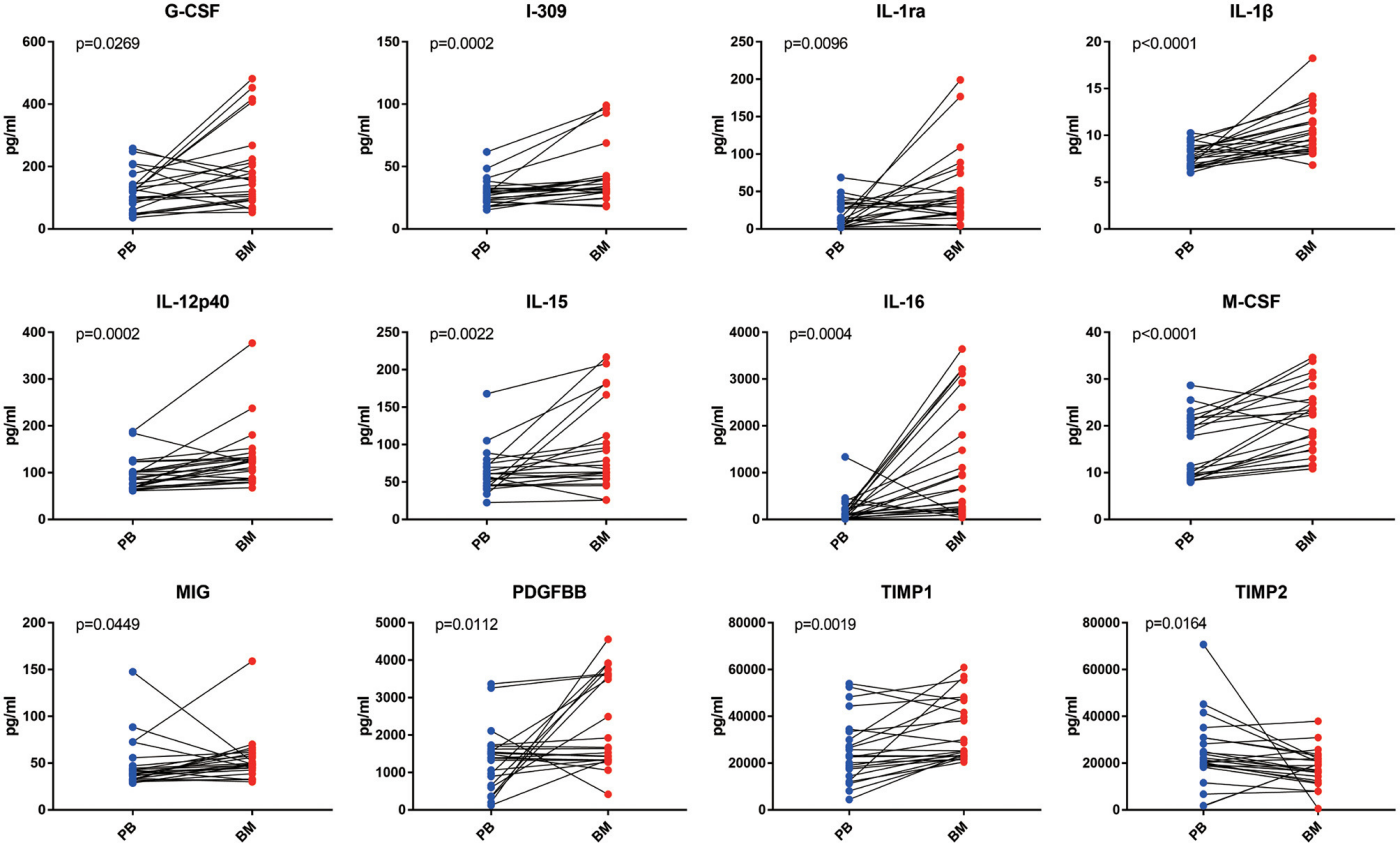
The analysis of inflammatory cytokine levels in PB and BM supernatant of MPN patients. BM supernatant levels of G-CSF, I-309, IL-1ra, IL-1β, IL-12p40, IL-15, IL-16, M-CSF, MIG, PDGF-BB, and TIMP-1 were significantly higher than those in PB (all *p* < 0.05). Only the level of TIMP2 in BM supernatant was lower than that in plasma.

In the paired comparative analysis, the BM levels of G-CSF [156.40 (95.34–221.20) pg/ml vs. 118.60 (66.13–141.60) pg/ml, *p* = 0.0269], I-309 [32.54 (29.52–40.70) pg/ml vs. 28.59 (22.24–32.76) pg/ml, *p* = 0.0002], IL-1ra [36.68 (20.03–68.73) pg/ml vs. 12.58 (2.89–34.59) pg/ml, *p* = 0.0096], IL-1β [9.92 (8.52–12.38) pg/ml vs. 7.75 (6.75–8.82) pg/ml, *p* < 0.0001], IL-12p40 [123.70 (87.91–133.40) pg/ml vs. 87.64 (68.39–102.10) pg/ml, *p* = 0.0002], IL-15 [75.59 (56.37–152.90) pg/ml vs. 60.81 (45.36–78.80) pg/ml, *p* = 0.0022], IL-16 [0.66 (2.02–22.55) × 10^2^ pg/ml vs. 1.09 (0.46–2.13) × 10^2^ pg/ml, *p* = 0.0004], M-CSF [25.24 (14.81–27.90) pg/ml vs. 14.67 (9.34–21.70) pg/ml, *p* < 0.0001], MIG [53.50 (43.15–59.50) pg/ml vs. 38.93 (33.26–46.59) pg/ml, *p* = 0.0449], PDGF-BB [15.86 (13.29–36.00) × 10^2^ pg/ml vs. 13.94 (7.18–16.85) × 10^2^ pg/ml, *p* = 0.0112] and TIMP-1 [25.23(22.95–45.56) × 10^3^ pg/ml vs. 23.01 (15.29–34.32) × 10^3^ pg/ml, *p* = 0.0019] were significantly higher than those in PB, except for TIMP-2 [18.09 (12.94–21.95) × 10^3^ pg/ml vs. 20.97 (18.73–30.23) × 10^3^ pg/ml, *p* = 0.0164] (Figure 2).

**Figure 3:**
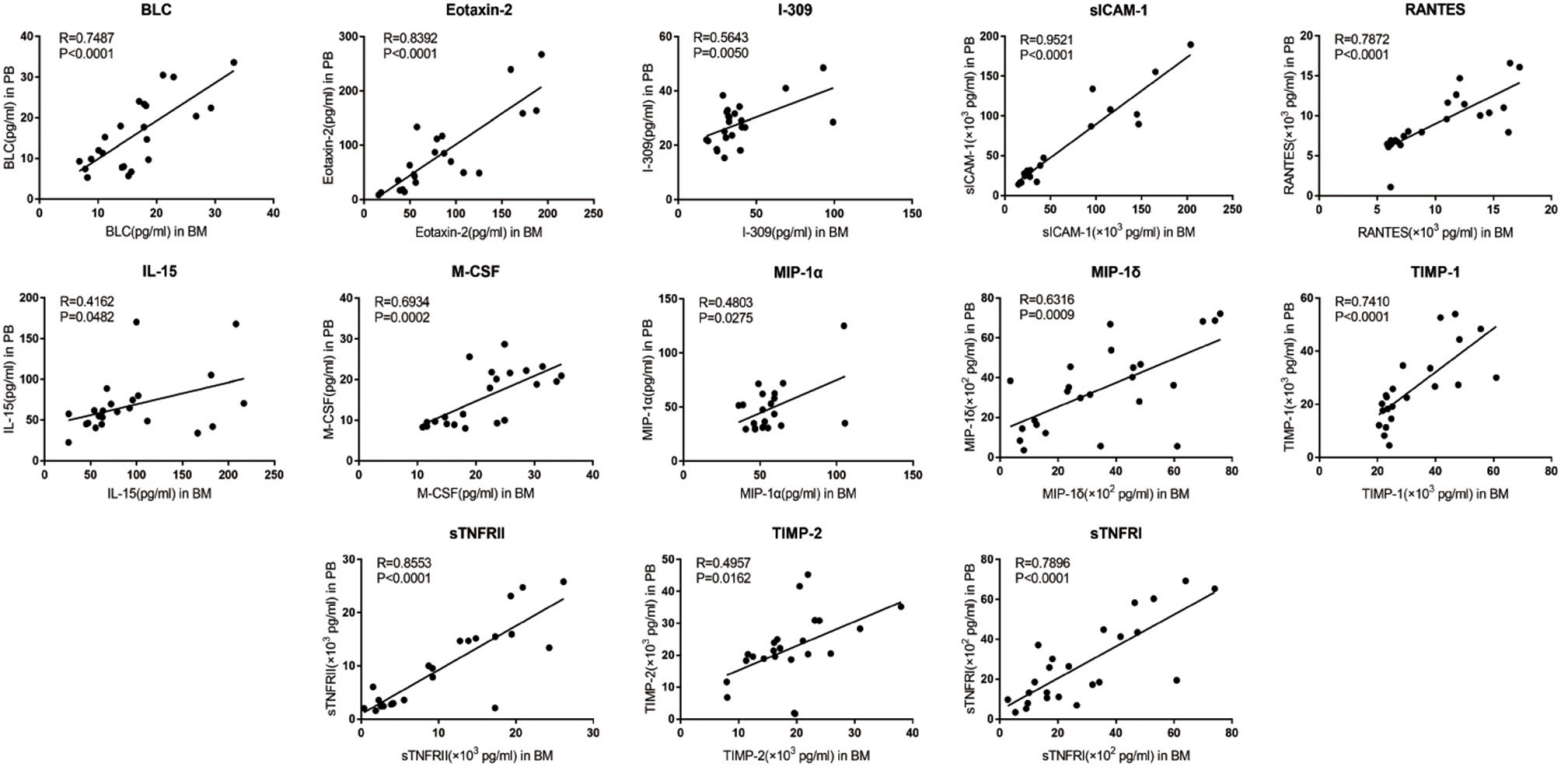
Correlation between PB and BM supernatant levels of inflammatory cytokine levels in MPN patients. BLC, Eotaxin-2, I-309, sICAM-1, IL-15, M-CSF, MIP-1α, MIP-1δ, RANTES, TIMP-1, TIMP-2, sTNFR I, and sTNFR II showed optimal linear correlation between PB levels and BM supernatant levels.

Of the 40 cytokines analyzed, levels of 13 cytokines were closely correlated between PB and BM [BLC (*R* = 0.7487, *p* < 0.0001), Eotaxin-2 (*R* = 0.8392, *p* < 0.0001), I-309(*R* = 0.5643, *p* = 0.005), sICAM-1(*R* = 0.9521, *p* < 0.0001), IL-15(*R* = 0.4162, *p* = 0.0482), M-CSF (*R* = 0.6934, *p* = 0.0002), MIP-1α (*R* = 0.4803, *p* = 0.0275), MIP-1δ (*R* = 0.6316, *p* = 0.0009), RANTES (*R* = 0.7872, *p* < 0.0001), TIMP-1 (*R* = 0.7410, *p* < 0.0001), TIMP-2 (*R* = 0.4957, *p* = 0.0162), sTNFRI (R = 0.7896, *p* < 0.0001), and sTNFRII (*R* = 0.8553, *p* < 0.0001)] (Figure 3).

### ELISA Validation of Cytokines as Circulating Biomarkers for MPN

**Figure 4:**
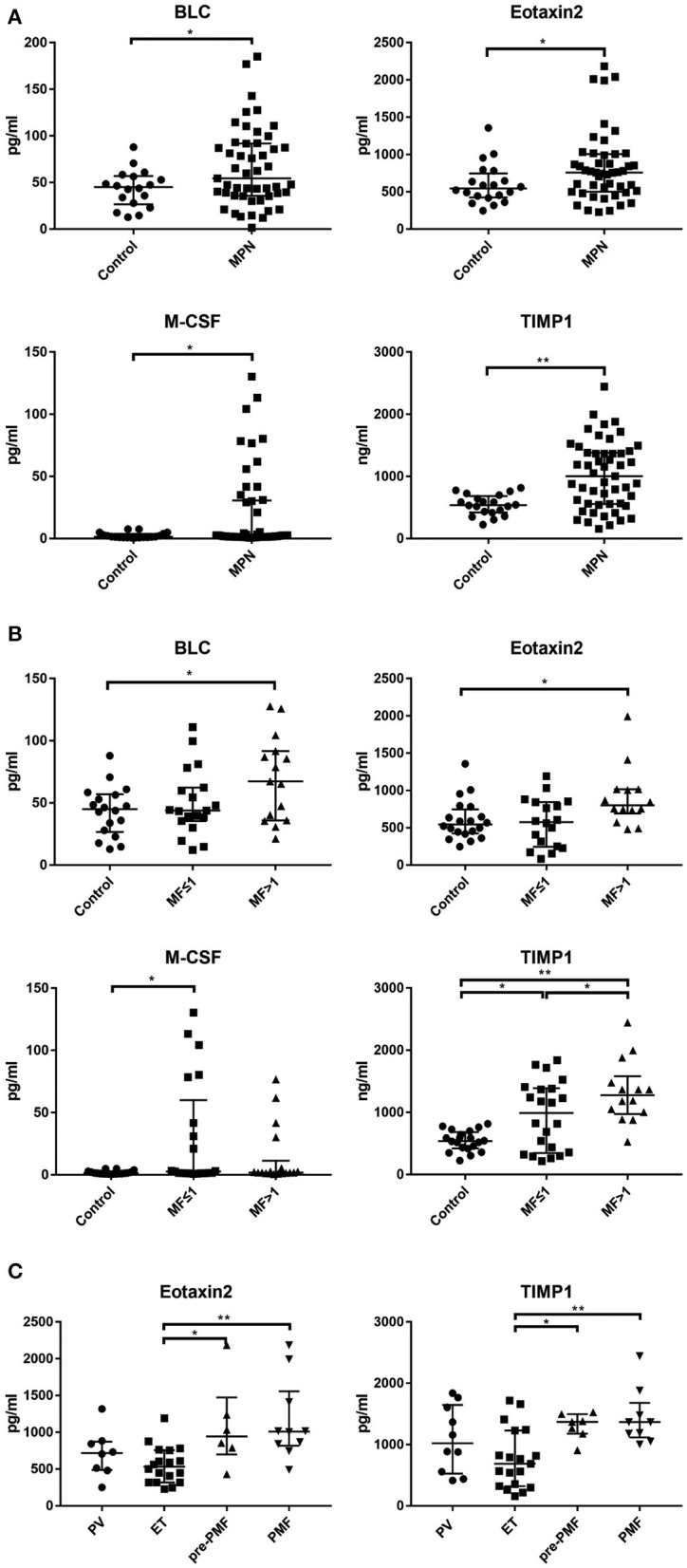
The levels of BLC, Eotaxin-2, M-CSF, TIMP-1 in PB in different clinical groups. **(A)** The plasma levels of BLC, Eotaxin-2, M-CSF, and TIMP-1 were significantly higher than health controls; **(B)** The plasma levels of BLC, Eotaxin-2, M-CSF, and TIMP-1 have significant differences among MF grade groups; **(C)** Eotaxin-2 and TIMP-1 levels have significant differences among disease subtypes. (^*^p < 0.05; ^**^p < 0.01).

An independent cohort of 52 MPN patients (12 with PV, 21 with ET, 6 with Pre-PMF and 13 with PMF) were enrolled for ELISA validation. Based on cytokine array result, 5 cytokines whose PB levels were significant different from the health controls (all *p* < 0.05) and also had optimal correlation between PB and BM supernatant levels (all *R* > 0.6, *p* < 0.05) were selected [BLC (*R* = 0.7487, *p* < 0.0001), Eotaxin-2 (*R* = 0.8392, *p* < 0.0001), M-CSF (*R* = 0.6934, *p* = 0.0002), TIMP-1 (*R* = 0.7410, *p* = 0.0030), sICAM-1 (*R* = 0.9521, *p* < 0.0001)] as potential candidates for further investigation. The results showed that the levels of BLC [54.44 (35.72–91.92) pg/ml vs. 45.09 (26.64–56.93) pg/ml, *p* = 0.0354], Eotaxin-2 [757.70 (500.30–1005.00) pg/ml vs. 545.00 (423.90–747.80) pg/ml, *p* = 0.0444], M-CSF [2.17 (1.35–30.64) pg/ml vs. 1.47 (0.96–3.67) pg/ml, *p* = 0.0263] and TIMP-1 [1004.00 (556.50–1381.00) ng/ml vs. 539.40 (421.60–683.70) ng/ml, *p* = 0.0003] in the plasma of MPN patients were significantly higher than that of the health controls, while the levels of sICAM-1 were not significantly different from the health controls [691.60 (575.70–921.90) vs. 647.00(476.00–801.70) ng/ml, *p* = 0.0618] (Table 1, Figure 4A).

**Table 1:** The validation results of plasma levels of BLC, Eotaxin2, M-CSF, TIMP1, and sICAM-1 in MPN and health control by ELISA.

	**Health control (*n* = 20)**	**MPN (*n* = 52)**	***P-*value**
BLC (pg/ml)	45.09 (26.64–56.93)	54.44 (35.72–91.92)	0.0354
Eotaxin2 (pg/ml)	545.00 (423.90–747.80)	757.70 (500.30–1005.00)	0.0444
M-CSF (pg/ml)	1.47 (0.96–3.67)	2.17 (1.35–30.64)	0.0263
TIMP1 (ng/ml)	539.40 (421.60–683.70)	1004.00 (556.50–1381.00)	0.0003
sICAM-1(ng/ml)	647.00 (476.00–801.70)	691.60 (575.70–921.90)	0.0618

### Several Cytokines Have Potential Application Value for the Diagnosis of MPN Subtypes

Comparing the plasma levels of cytokines grouped according to the grade of MF, it was found that the levels of BLC [67.25 (35.99–91.68) pg/ml vs. 45.09 (26.64–56.93) pg/ml, *p* = 0.0218], Eotaxin-2 [800.20 (692.40–1015.00) pg/ml vs. 545.00 (423.90–747.80) pg/ml, *p* = 0.0498], and TIMP-1 [1277.00 (975.00–1580.00) ng/ml vs. 539.40 (421.60–683.70) ng/ml, *p* < 0.0001] in the MF > 1 group were significantly higher than those of health control; the levels of M-CSF [2.68 (1.35–60.08) pg/ml vs. 1.35 (0.91–2.34) pg/ml, *p* = 0.040] and TIMP-1 [989.60 (347.30–1388.00) ng/ml vs. 539.40(421.60–683.70) ng/ml, *p* = 0.0113] in group MF ≤ 1 were also significantly higher than those of health control. In addition, the level of TIMP-1 in group MF > 1 was significantly higher than that of MF ≤ 1 group [1277.00 (975.00–1580.00) ng/ml vs. 989.60 (347.30–1388.00) ng/ml, *p* = 0.0359] (Figure 4B).

The level of TIMP-1 in the ET group was significantly lower than that of the pre-PMF group [685.20 (322.20–1229.00) ng/ml vs. 1369.00 (1175.00–1497.00) ng/ml, *p* = 0.0221] and PMF group [685.20 (322.20–1229.00) ng/ml vs. 1365.00 (1115.00–1681.00) ng/ml, *p* = 0.0006]; the level of Eotaxin-2 in ET group was significantly lower than that of pre-PMF group [531.4 (317.9–756.6) pg/ml vs. 942.4 (699.3–1,474) pg/ml, *p* = 0.0393] and PMF group [531.40 (317.90–756.60) pg/ml vs. 1010.00 (818.00–1556.00) pg/ml, *p* = 0.0030]; BLC, M-CSF, and sICAM-1 showed no significant difference between the disease subtype groups (Figure 4C).

There were no significant differences in the comparation of cytokine levels between different driver gene mutation groups (*JAK2, MPL*, or *CALR* mutation).

## Discussion

Ph-MPNs are a series of acquired clonal hematopoietic malignancies characterized by accumulation of matured myeloid cells in the BM and PB. Mutations of *JAK2, CALR*, and *MPL* genes are the key drivers in the pathogenesis of MPN (1). Recent studies revealed that persistent chronic inflammation is also a major “driver” that contributes to the development and progression of MPN, which affects the severity of the disease and is related to complications (8). As important mediators of intercellular communication, a large number of cytokines act in autocrine, paracrine and endocrine manners in local microenvironment to play an intricate network regulation (9). However, most of the studies have only focused on the inflammatory cytokine profile in the PB of MPN patients. Studies of cytokines in the BM have been limited to co-cultivation experiments of BM-derived cells or cell lines *in vitro* (10, 11).

The BM niche, which is described as a relatively barriered tumor microenvironment of hematological neoplasms, has been shown to play a major role in maintaining the proliferation and differentiation of hematopoietic stem cells (HSCs) (12). There is emerging evidence that the inflammatory cytokine storm inside the BM niche might be the main trigger for MF and even AML transformation during the progression of MPN, which seriously compromise patients' life quality and cause inferior survival (9, 13, 14).

In present study, we simultaneously examined the levels of 40 inflammatory cytokines in the BM supernatant and PB plasma of MPN patients using protein microarray. Compared to healthy controls, the plasma levels of 10 cytokines of MPN patients, including BLC, Eotaxin-2, IL-7, IL-8, IL-13, IL-10, M-CSF, MIP-1β, sICAM-1, and TIMP-1, were remarkably elevated. This finding is consistent with previous researches, which indicates that the inflammatory status is involved in the pathophysiology of MPN (2, 15, 16). The levels of 11 cytokines (G-CSF, I-309, IL-1ra, IL-1β, IL-12p40, IL-15, IL-16, M-CSF, MIG, PDGF-BB, and TIMP-1) in the BM supernatant were much higher than those in the PB, suggesting a more intense ongoing inflammatory process in the BM. To our knowledge, this is the first study that directly revealed the inflammatory hallmark in the BM microenvironment of MPN.

Current study also found that cytokine levels are not always correlate to each other between plasma and BM supernatant. A variety of cells in the BM cavity including hematopoietic cells, mesenchymal cells, macrophages, fibroblasts, osteoblasts, as well as various matrix components such as adhesion molecules, chemokines, cytokines, and soluble or membrane-bound factors present in the BM stroma form a relatively barriered and distinguished microenvironment so called BM niche (17, 18). Dysregulation of the BM microenvironment has been proved to be an important step in the development of many myeloid malignancies (19). Hematopoietic stem cells regulators, such as sICAM-1, is BM niche dependent (12). Abnormally proliferating neoplasm cells in MPN might stimulate normal hematopoietic cells and stromal cells in the BM niche to secrete more pro-inflammatory factors, which further aggravated the inflammation inside the niche (7, 20). To this regard, our results also suggest that cautions should be exerted when interpreting of the inflammatory situation inside the BM by merely PB metrics.

Furthermore, a panel of circulating cytokines which includes BLC, M-CSF, Eotaxin-2, and TIMP-1, was identified to have the capability of reflecting inflammatory status in BM niche, which could be potentially served as biomarkers for the diagnosis of MPN subtypes and the prediction of fibrosis progression.

Specifically, BLC, also known as CXCL13, has been reported to be highly expressed in many autoimmune diseases, leading to abnormal lymphocyte recruitment and tissue damage (21). In current study, elevated BLC level was identified in the MF>1 group, which may be related to the involvement of BLC in osteosclerosis. Studies have found that BLC could promote the differentiation of mesenchymal stem cells into osteoblasts by inhibiting miR-23a expression and up-regulating the level of ERK1/2 kinase (22, 23).

M-CSF, macrophage megakaryocyte colony stimulating factor, can promote the differentiation and proliferation of monocytes and macrophages. It is known that M-CSF can upregulate MCP-1 and MIP-1, resulting in the infiltration of monocytes in liver injury area and aggravating inflammation (24). Our data showed that M-CSF levels in MPN were significantly higher than health controls. MIG and MIP-1β, which is related to macrophage proliferation and activation, also showed higher expression in MPN, suggesting that there may be a macrophage dysregulation similar to liver fibrosis.

Another cytokine, Eotaxin-2, or CCL24, is an eosinophil chemokine. Eotaxin-2 enhanced the proliferation of fibroblasts and collagen synthesis, leading to airway remodeling in asthma patients (25). High level of Eotaxin-2 was found in the bronchoalveolar fluid of patients with idiopathic pulmonary fibrosis, which was considered to be an important contributor for fibrosis formation (26). In line with previous studies, the level of Eotaxin-2 was significantly increased in MPN, especially in the MF>1 group.

The most valuable molecule for discriminating MPN subtypes and monitoring disease progression the present study revealed is TIMP-1, one of the members of the TIMP glycoprotein family. As a natural inhibitor of matrix metalloproteinases (MMPs), increased TIMP-1 can inhibit the degradation of MMPs on the extracellular matrix including collagen fibers (27). Additionally, as an important cell development regulatory protein, TIMP-1 was also revealed to has the effect of promoting proliferation and inhibiting apoptosis (28). We found that different MF grading groups of MPN had quite distinct TIMP-1 levels, and significantly elevated plasma TIMP-1 levels were associated with high grade MF. Ho et al. also found that patients with PMF, instead of those with either PV or ET, displayed significantly higher levels of TIMP-1 than that of the normal control group. The author therefore believed that TIMP-1 played an important role in the pathogenesis of myelofibrosis (29). Among the subtypes of Ph^−^MPNs, the early stage of PMF (WHO newly named Pre-PMF) and ET are both characterized by thrombocytosis and rarely specific clinical features, which bring challenges for differential diagnosis. Compared to ET, Pre-PMF has a much faster progression rate to PMF or even AML, requiring more aggressive treatment options.

Our results showed that the plasma levels of TIMP-1 and Eotaxin-2 in the PMF and prePMF groups were significantly higher than those in the ET group, respectively, suggesting that these two cytokines have potential diagnostic values in the early identification of the disease subtypes of MPN. MF can evolve from PV or ET (called post-PV/ET-MF), which is a serious adverse event during the chronic progression of MPN. The severity of fibrosis is closely related to poor prognosis and short survival. Usually, MPN patients need to perform BM biopsy, an invasive operation, to determine whether MF has occurred during long-term follow-up. Here we propose that TIMP-1 in PB plasma, could be used as a non-invasive predictor of MF, guiding early judgment of disease progression, and indicating the time point for BM biopsy.

### Limitations

To our knowledge, the present study is the first to examine inflammatory cytokine levels in BM niche of MPN patients. However, findings of the study should be viewed and interpreted in the light of its limitations. As this is an observation study, which is inherent to all known associated shortcomings, future functional studies are warranted to investigate underling mechanism of MF prompting and clonal evolution. Besides, cytokines examined in the study only represent a small fraction of hundreds or thousands of molecules involved in the related inflammatory processes. Also, the evidence could be much solid if the study had a health BM donor group to compare with BM niche inflammation. In addition, the relatively small sample size might have hindered generalization of the key findings. Large-scale, multi-center studies are needed to validate current results, and to further explore the association between these inflammation mediators and the development of MPN. Lastly, the study describes only one time-point results statically. As the inflammatory responses involved in disease progression are complex, findings should be further evaluated dynamically.

## Conclusion

Cytokine profiling among Ph-MPN patients revealed a more severe inflammatory state inside the BM niche for the first time. A panel of cytokines in PB was identified to be capable of assessing the inflammation status of BM microenvironment in MPN. Specifically, BLC, Eotaxin-2, M-CSF, and TIMP-1 could potentially aid in the diagnosis and prediction of fibrosis progress of MPN subtypes as non-invasive biomarkers. Future studies are warranted to investigate the cytokines associated signaling that could shed light on the associated molecular mechanisms.

## Data Availability Statement

The original contributions presented in the study are included in the article/Supplementary Material, further inquiries can be directed to the corresponding author/s.

## Ethics Statement

The studies involving human participants were reviewed and approved by our Institutional (Zhongshan Hospital, Fudan University) Review Board. The patients/participants provided their written informed consent to participate in this study.

## Author Contributions

PC, BW, and YC conceived the study. PC, JC, FL, and YC performed the literature review and drafted and revised the manuscript. HaC and YC contributed to the critical revision of the manuscript. YZ, FH, ZM, HeC, YK, LC, LJ, and LS performed the experiments and analyzed data. All authors read and approved the final manuscript.

## Conflict of Interest

The authors declare that the research was conducted in the absence of any commercial or financial relationships that could be construed as a potential conflict of interest.

## Abbreviations

BLC: B lymphocyte chemoattractant
Eotaxin: Eosinophil chemotactic protein
G-CSF: Granulocyte colony stimulating factor
M-CSF: Macrophage colony stimulating factor
GM-CSF: Granulocyte- macrophage colony stimulating factor
IFN: Interferon
ICAM: Intercellular adhesion molecule
MCP: Monocyte chemotactic protein
MIP: Macrophage inflammatory protein
RANTES: Regulated on activation Normal T cell expressed and secreted
MIG: Monokine induced by gamma interferon
IL: Inerleukin
TIMP: Tissue inhibitor of metalloproteinases
PDGF-BB: Platelet derived growth factor subunit B
TNF: Tumor necrosis factor
sTNFR: Soluble tumor necrosis factor receptor.
