# Supplementary material for: Cytokine Consistency Between Bone Marrow and Peripheral Blood in Patients With Philadelphia-Negative Myeloproliferative Neoplasms
Source: Front Med (Lausanne). 2021 Jun 24;8:598182. doi: 10.3389/fmed.2021.598182 (PMC8264196; doi:10.3389/fmed.2021.598182)
Supplement: Supplementary file 1 [file Table_1.docx]

**supplementary table 1**

Results of inflammation-associated cytokines in Plasma and BM supernatant by protein antibody array

|  | **Health Control（n=12）** | **MPN（n=24）** | |
| --- | --- | --- | --- |
|  | PB(pg/ml) | PB (pg/ml) | BM (pg/ml) |
| BLC | 10.17(8.97-11.34) | 14.66(7.95-22.95)^#※^ | 16.36(10.87-18.51)^※^ |
| Eotaxin-1 | 94.53(88.93-105.70) | 92.98(56.13-183.60) | 120.40(88.81-167.2) |
| Eotaxin-2 | 40.96(30.25-52.62) | 66.47(32.63-152.30) ^#^ | 67.34(42.49-121.00) |
| G-CSF | 109.70(99.08-130.50) | 118.60(66.13-141.60) | 156.40(95.34-221.20) |
| GM-CSF | 60.68(44.09-76.57) * | 82.31(16.88-262.10） | 107.50(69.04-193.70) |
| I-309 | 83.31(63.73-92.99) | 28.59(22.24-32.76) ^#※^ | 32.54(29.52-40.70) ^※^ |
| sICAM-1×10^3^ | 21.94(14.44-32.21) | 29.78(24.94-98.98) ^#^ | 26.83(22.31-95.93) |
| IFN-γ | 63.83 (42.57-610.60) | 138.20 (38.53-407.50) | 196.90(50.31-325.20) |
| IL-1α | 41.79(27.83-51.81) | 48.92 (10.00-192.30) | 56.28(10.00-178.50) |
| IL-1β | 59.00(49.86-68.50) | 7.75 (6.75-8.82) ^#※^ | 9.92(8.52-12.38) ^※^ |
| IL-1ra | 133.20(99.74-184.20) | 12.58 (2.89-34.59) ^#※^ | 36.68(20.03-68.73) ^※^ |
| IL-2 | 14.45(10.83-220.20) | 91.54(39.90-137.70) | 135.71(53.33-259.10) |
| IL-4 | 35.62(21.67-340.80) | 106.74(37.52-228.60) | 148.2(41.25-228.90) ^※^ |
| IL-5 | 157.40(124.90-210.50) | 37.98 (24.57-56.14) ^#※^ | 64.42(31.73-107.50) |
| IL-6 | 66.97(53.47-90.00) | 64.35(36.19-105.80) | 87.33(32.75-190.40) |
| IL-6 R×10^2^ | 48.00(44.04-185.62) | 88.24 (76.82-98.72) | 82.58 (75.62-92.73) |
| IL-7 | 96.10(74.66-127.40) | 318.10(100.90-727.00) ^#※^ | 454.00(224.20-762.70)^※^ |
| IL-8 | 5.71(4.02-7.67) | 14.15 (7.60-32.83) ^※^ | 33.34(10.80-44.40) ^※^ |
| IL-10 | 18.74(14.86-47.03) | 37.82(21.82-54.32) ^#^ | 39.77(22.15-85.77) ^※^ |
| IL-11×10^2^ | 8.70(5.51-13.06) | 3.27(1.89-5.06) ^#^ | 8.21(3.13-12.40) |
| IL-12p40 | 74.68(37.99-96.05) | 87.64 (68.39-102.10) ^※^ | 123.70(87.91-133.40) |
| IL-12p70 | 20.70 (12.11-38.20) | 5.91 (4.61-13.81) ^#※^ | 7.61(4.97-23.53) ^※^ |
| IL-13 | 14.83(10.68-33.83) | 35.98(19.62-74.48)^#※^ | 44.76(25.35-77.93)^※^ |
| IL-15 | 60.22(30.06-74.11) | 60.81(45.36-78.80) ^※^ | 75.59(56.37-152.90) |
| IL-16×10^2^ | 1.62(0.93-1.94) | 1.09 (0.46-2.13) ^※^ | 0.66 (2.02-22.55) |
| IL-17A | 30.15(24.07-38.85) | 43.61(20.67-61.23) ^※^ | 49.84(29.06-124.30) |
| MCP-1 | 298(212.4-374.4) | 192.11(90.93-463.40) | 254.07(129.30-276.20) |
| M-CSF | 9.65(6.37-12.97) | 14.67(9.34-21.70) ^#※^ | 25.24(14.81-27.90) ^※^ |
| MIG | 62.42(28.65-71.52) | 38.93(33.26-46.59) | 53.50(43.15-59.50) |
| MIP-1α | 88.48(23.90-126.20) | 49.58(31.97-69.26) | 117.09(47.38-64.73) |
| MIP-1β | 11.40(4.36-82.85) | 60.36 (25.85-116.20) ^#※^ | 127.66(57.31-166.60) ^※^ |
| MIP-1δ×10^2^ | 28.93(23.01-39.98) | 34.19(14.93-46.38) | 32.86(13.34-48.36) |
| PDGF-BB×10^2^ | 16.44(14.72-31.08) | 13.94(7.18-16.85) | 15.86(13.29-36.00) |
| RANTES×10^3^ | 7.31(3.24-14.77) | 8.00(6.53-11.59) | 9.74 (6.18-13.53) |
| TIMP-1×10^3^ | 12.16(7.38-18.41) | 23.01(15.29-34.32)^#※^ | 25.23(22.95-45.56) ^※^ |
| TIMP-2×10^3^ | 19.60(15.73-23.26) | 20.97 (18.73-30.23) | 18.09(12.94-21.95) |
| TNFα | 96.08(74.85-126.70) | 104.33(47.65-284.70) | 144.20(70.55-240.20) |
| TNFβ | 87.08(69.49-106.60) | 109.70(49.39-282.20) | 253.80(53.78-475.7) |
| sTNFRⅠ×10^2^ | 19.67(10.76-45.39) | 18.99 (10.76-42.94) | 22.07(12.36-45.26) |
| sTNFRⅡ×10^2^ | 30.23(19.53-166.73) | 69.57 (27.52-150.58) | 89.64(26.91-173.18) |

#：*p<*0.05 ,compared with the BM supernatant; ※：*p<*0.05 compared with the health controls.
