# Supplementary material for: Cytokine Consistency Between Bone Marrow and Peripheral Blood in Patients With Philadelphia-Negative Myeloproliferative Neoplasms
Source: Front Med (Lausanne). 2021 Jun 24;8:598182. doi: 10.3389/fmed.2021.598182 (PMC8264196; doi:10.3389/fmed.2021.598182)
Supplement: Supplementary file 2 [file Table_2.docx]

Supplementary table 2.

Correlation analysis between the plasma levels of cytokines and the complete blood count parameters in MPN patients. (n=52)

|  | RBC | PLT | WBC |
| --- | --- | --- | --- |
| BLC | R=-0.1608  *p*=0.2753 | R=-0.1545  *p*=0.2943 | R=0.0895  *p*=0.5454 |
| M-CSF | R=0.3234  *p*=0.1904 | R=-0.2635  *p*=0.2908 | R=-0.0275  *p*=0.9137 |
| TIMP1 | R=0.0141  p=0.9224 | R=0.0807  *p*=0.5777 | R=-0.0039  *p*=0.9785 |
| Eotaxin2 | R=0.0691  *p*=0.6372 | R=0.2038  *p*=0.1602 | R=0.1861  *p*=0.2004 |

Table The p value and r value are obtained by linear regression of cytokine levels with RBC, PLT, and WBC respectively. The plasma levels of BLC, M-CSF, TIMP-1 and Eotaxin-2 have no correlation with the levels of RBC, PLT and WBC respectively.
